# Inter-Semispinalis Plane Block Versus General Anesthesia for Postoperative Analgesia in Posterior Cervical Spine Surgery: A Randomized Controlled Trial

**DOI:** 10.5812/aapm-143369

**Published:** 2024-02-16

**Authors:** Atef Mohamed Mahmoud, Mohammed Awad Alsaied, Safaa Gaber Ragab, Youmna Ahmed Abdelfattah, Omer Sayed Farghaly, Mohamed Ahmed Shawky

**Affiliations:** 1 Faculty of Medicine, Fayoum University, Fayoum, Egypt

**Keywords:** Posterior Cervical Spine, Inter-Semispinalis Plane (ISPB) Block, Analgesia, Pain Management, opioids, Non-steroidal Anti-inflammatory Drugs (NSAIDs), Fentanyl, Visual Analog Scale (VAS), Rescue Analgesia, Complications, Surgeon Satisfaction

## Abstract

**Background:**

Postoperative pain management is crucial for improving patient outcomes following posterior cervical spine surgery. Opioids are effective but carry a risk of respiratory depression. Non-steroidal anti-inflammatory drugs (NSAIDs) are commonly used but may not provide adequate pain relief and have potential complications. The inter-semispinalis plane (ISPB) block is a novel technique for postoperative analgesia in cervical spine surgery.

**Objectives:**

This study aims to evaluate and compare the efficacy of the ISPB with general anesthesia in terms of analgesia, postoperative Visual Analog Scale (VAS) pain scores, patient-surgeon satisfaction levels, and the occurrence of postoperative complications.

**Methods:**

This double-blind, randomized controlled trial was blinded to both the patient and the assessor. Fifty adult patients (18 - 60 years old) undergoing elective posterior cervical spine surgery were enrolled. The participants were divided into 2 groups: The ISPB group (receiving bilateral ultrasound-guided ISPB at the C5 level) and the control group (receiving general anesthesia only), with each group comprising 25 patients. The study assessed intraoperative fentanyl use, postoperative VAS pain levels, the need for rescue analgesia, and complications.

**Results:**

The ISPB group showed significantly lower intraoperative fentanyl consumption (median 100 vs. 100 - 150 μg, P = 0.022) and lower postoperative pain scores at 1, 8, 12, and 48 hours (P = 0.016, 0.009, 0.005, 0.016). Additionally, the ISPB group required less postoperative pethidine (20% vs. 64%, P = 0.002) and had a longer delay before requesting pethidine (hazard ratio 0.215, P = 0.001). Surgeon satisfaction was significantly higher in the ISPB group (P = 0.003). These results suggest that the ISPB can effectively reduce pain and analgesic requirements.

**Conclusions:**

The ISPB is an effective analgesic technique for posterior cervical spine surgery, reducing opioid consumption, providing better pain control, and enhancing surgeon satisfaction without increasing complications. This approach has the potential to improve postoperative care and patient outcomes in this surgical population.

## 1. Background

Posterior cervical spine surgery, often performed on older individuals with significant comorbidities, is one of the most painful surgical operations. Anesthesiologists face a unique challenge in managing pain following these surgeries (1). Considering the surgical process, clinical state, and patient histories, the posterior approach to cervical spine surgery presents a distinct difficulty for anesthesiologists. This surgery is ranked among the 6 most painful out of 179 assessed surgical procedures, underscoring the challenges in pain management for this patient population. Many patients requiring spine surgery are also overweight, have substance use disorders, or other comorbidities related to aging (2). Postoperative discomfort is common following posterior cervical spine surgery, impeding early mobility and rehabilitation (3). Patients experiencing this consequence endure severe pain, which slows their recovery and increases morbidity. Thus, effective pain management techniques are crucial to enhance postoperative care for these patients (4, 5) Although opioid analgesics are beneficial, their use risks respiratory depression. Non-steroidal anti-inflammatory drugs (NSAIDs) are frequently used as initial treatments for pain (6), yet they may not provide sufficient pain relief. Notably, high doses of NSAIDs have been associated with nonunion issues after spine fusion procedures, emphasizing the importance of well-formulated analgesics in such cases (7). The inter-semispinalis plane (ISPB) block, an anatomy-based modified technique involving local anesthetic injection into the fascial channel between the semispinalis cervicis and semispinalis capitis muscles (8), has limited established efficacy and safety due to its application in only a few case series. The effectiveness and outcomes of the ISPB as an opioid-sparing analgesic approach for cervical spine surgery remain uncertain, indicating a knowledge gap in this specific surgical context.

## 2. Objectives

This study aims to evaluate and compare the effectiveness of the ISPB in terms of analgesia, postoperative Visual Analog Scale (VAS) pain scores, patient-surgeon satisfaction levels, and the occurrence of postoperative complications compared to general anesthesia.

## 3. Methods

### 3.1. Ethics Approval and Registration

This study involved 50 patients who were randomized into 2 groups ISPB group and control group) in a 1:1 ratio, with 25 patients in each group. There were no patients lost to follow-up, no exclusions, and all were analyzed as part of the study. Following approval from the local Institutional Review Board and the local Institutional Ethics Committee (ethical committee number: M644 and ClinicalTrials.gov number: NCT06003933), the study was conducted at Fayoum University Hospital. A randomized controlled trial with double-blinding was the chosen design. Before enrollment and random assignment, eligible patients were required to provide comprehensive informed consent.

### 3.2. Inclusion Criteria

The study included adult patients (ASA physical status I-II) undergoing elective posterior cervical spine surgery. The cohort consisted of both male and female patients aged 18 to 60 years. As part of the pre-anesthetic consultation, each patient was thoroughly informed about the study's procedures, the intricacies of the ISPB, and the use of the VAS for pain measurement.

### 3.3. Exclusion Criteria

Patients with a history of cervical disc surgery or fixation, mental health disorders, substance abuse, or contraindications to regional anesthesia (such as local infection or coagulation abnormalities) were excluded from the study.

### 3.4. Anesthesia Procedure and Interventions

A complex randomization technique utilizing computer-generated list sequences was applied to the patients. Subsequently, with the highest level of secrecy maintained, they were discreetly divided into 2 separate cohorts. This division was facilitated using sealed, opaque envelopes. Group C is tasked with influencing the administration of exclusive general anesthesia and operates from one corner. On the other hand, the ISPB group is executing its intricate strategy. This strategy includes the precise placement of bilateral ultrasound-guided ISPB targeting the C5 vertebra, coupled with the infusion of a meticulously prepared mixture containing 10 mL of 0.25% bupivacaine and an equal amount of Xylocaine 1%. This elaborate effort aims to mitigate potential toxicity concerns. Each participant is administered an oral preoperative dose of midazolam, precisely calculated at 0.5 mg/kg, to be taken 30 minutes before their surgery begins. However, prior to this administration, as part of our comprehensive preoperative evaluation, we initiate a detailed analysis of their preoperative VAS scores. The establishment of intravenous (IV) access, a 5-lead ECG, pulse oximetry, non-invasive blood pressure monitoring, capnography, and other comprehensive monitoring procedures are all meticulously executed as our participants stand on the brink of their medical journey. The process of administering general anesthesia unites all participants in a cohesive procedure. The initial phase involves a skillfully administered IV induction with 2 mg/kg of propofol and 1 g/kg of fentanyl, which leads to a state of drug-induced sedation. This is followed by a critical dose of 0.5 mg/kg of atracurium, serving as the pivotal moment, facilitating orotracheal intubation and marking the participants' transition into anesthetic unconsciousness. During the maintenance phase of anesthesia, isoflurane inhalation is carefully adjusted between 1.2% and 1.5%, alongside a 50% - 50% mixture of oxygen and air. This phase is characterized by the gradual administration of atracurium, which ensures continued muscle relaxation, adding further intricacy to the procedure. Interestingly, while under the influence of general anesthesia, individuals in the selected group undergo the complex application of ISPB. In a seamless transition, another anesthesiologist, proficient in ultrasound-guided nerve block techniques yet not further involved in the study, steps in to replace the treating anesthesiologist, ensuring the continuity of care. In contrast, the journey of the control group is marked by the superficial ritual of skin preparation and a brief ultrasonic probe examination to assess the block size, devoid of the deeper complexity observed in the ISP group. A fifteen-minute interval was allotted before the commencement of the main procedure, providing an opportunity for reflection and analysis. The participants received fentanyl at a dosage of 0.5 μg/kg intravenously, a nuanced intervention intended to modulate their physiological state in cases where signs of inadequate anesthesia were observed, indicated by a disconcerting increase of more than 20% in heart rate or mean arterial blood pressure. Throughout this period, the meticulous monitoring of fentanyl administration served as a vigilant overseer, closely attuned to the fluctuations within this intricate medical trial.

### 3.5. Block Procedure Technique

The duty of performing the ultrasound-guided regional anesthetic technique was entrusted to the same anesthesiologist who had refined their expertise in this particular field. They embarked on this intricate procedure with a transversely oriented linear probe (SonoSite Edge, Bothell, Washington) to delineate the five-layered posterior cervical muscles located at the C5 vertebral level. The journey began at the C7 spinous process, from where the probe meticulously navigated upwards to the fifth cervical vertebra. This ultrasound imaging took place after the anesthesia induction and once the patient was positioned in a prone stance prior to making the skin incision. In preparation for this critical operation, the injection site was subjected to thorough aseptic preparation, facilitating the insertion of a 22-G, 50-mm block needle (Visioplex, Vygon). This slender yet formidable instrument was poised for in-plane insertion through the skin. The procedure evoked a deep sense of anatomical exploration as it delved into the intricate layers of the fascial plane nestled between the semispinalis cervicis and semispinalis capitis muscles with unwavering precision. The peak of this intricate operation was the administration of a substantial volume (20 mL) of the 0.25% bupivacaine solution. This critical step was taken only after carefully ensuring that there was no blood aspiration, thus preserving the safety and integrity of the procedure (8). The complex surgical procedure was concluded, and all anesthesia treatments were immediately ceased. Extubation was promptly carried out as soon as the patient demonstrated sufficient spontaneous respiratory function. This rapid recovery was facilitated by the precise administration of 0.05 mg/kg of neostigmine, combined with 0.02 mg/kg of atropine to achieve an effective reversal of the anesthesia. Following institutional guidelines, the patient was administered 1 g of intravenous paracetamol upon the completion of the surgery, a regimen to be continued every 12 hours thereafter. Upon the patient's arrival in the Post-Anesthesia Care Unit (PACU), vigilant monitoring of systolic, diastolic, and mean arterial blood pressure, heart rate, respiration rate, oxygen saturation, and SpO2 was initiated to closely observe the patient's physiological status. The complex VAS was employed as the judge to assess the effectiveness of the analgesic intervention, with its scale extending from 0, denoting no pain, to 100, representing the most excruciating pain imaginable. The postoperative assessment was conducted at intervals of 30 minutes, 1 hour, 2 hours, 4 hours, 6 hours, 8 hours, 12 hours, 18 hours, and 24 hours after the surgery, documenting the evolving narrative of the patient's experience. In the event that the VAS reached the critical threshold of 40, indicative of significant pain, an intervention with pethidine at a dosage of 0.5 mg/kg would be initiated. This intervention served as a crucial measure for pain relief, with the timing of the first request for this analgesic marking a key moment in the patient's postoperative journey. Bupivacaine levels in the blood were monitored closely, with measurements taken immediately after its administration and subsequently at 2, 4, 6, and 12 hours post-procedure. Throughout the surgical procedures and the following period, diligent observation and documentation of a broad spectrum of potential complications were maintained. Local anesthetic toxicity, the risk of hypotension (low blood pressure), the occurrence of nausea, and episodes of vomiting were among the complications monitored. Furthermore, we paid close attention to and documented any neurological symptoms, such as numbness, weakness, or other potential neurological concerns. To measure patient satisfaction - a crucial yet often elusive aspect of our study - a three-level scale was employed and meticulously evaluated. Patients who left feeling extremely satisfied were assigned a score of "three," while those who were moderately pleased received a "two." Regrettably, individuals expressing the lowest level of satisfaction, often experiencing challenging recoveries, were allocated a score of "one."

### 3.6. Primary Outcome Measures

Ingestion of pethidine within the first 48 hours after procedure.

### 3.7. Secondary Outcome Measures

The VAS score at 48 hours after surgery, the amount of fentanyl used during surgery, the incidence of postoperative nausea and vomiting, the extent of pruritus development, and any identified issues were all evaluated. These assessments were conducted by a professional anesthesiologist who was unaware of the group allocation.

### 3.8. Statistical Analysis and Sample Size Estimation

We conducted our statistical study using IBM Co.'s SPSS application for Windows version 28, based in Armonk, New York, United States. Additionally, the Kaplan-Meier curve and survival analysis were performed using MedCalc Statistical Software version 20 from MedCalc Software in Ostend, Belgium. Parametric quantitative data, expressed as means and standard deviations (SD), were analyzed using the unpaired student *t*-test. For non-parametric quantitative data based on medians and interquartile range (IQR), we employed the Mann-Whitney test for comparison. The analysis and presentation of categorical data involved calculating frequencies and percentages. To perform this analysis, we applied either Fisher's exact test or the chi-square test as appropriate.

For the evaluation of anesthesia's impact on the time it took for patients to request their first rescue analgesia, we employed Kaplan-Meier survival analysis, supported by the Log-rank test.

To determine the sample size for our study, we utilized the G*Power 3.1.7.9 tool from Heinrich-Heine-Universitat Düsseldorf in Düsseldorf, Germany. We set our two-tailed P-value cutoff for statistical significance at 0.05. Following Mostafa et al.'s observations, our objective was to detect a difference in VAS scores of 10 points with an effect size of 0.8. Based on these parameters, we estimated that achieving a 90% power would necessitate a minimum of 23 patients per group to identify a significant difference in postoperative pethidine intake at a significance level of 0.05. To account for possible withdrawals, we chose to recruit 25 patients in each group. We employed a two-sample independent, two-sided *t*-test to calculate the sample size, emphasizing our commitment to conducting precise research (9).

## 4. Results

**Figure 1.**
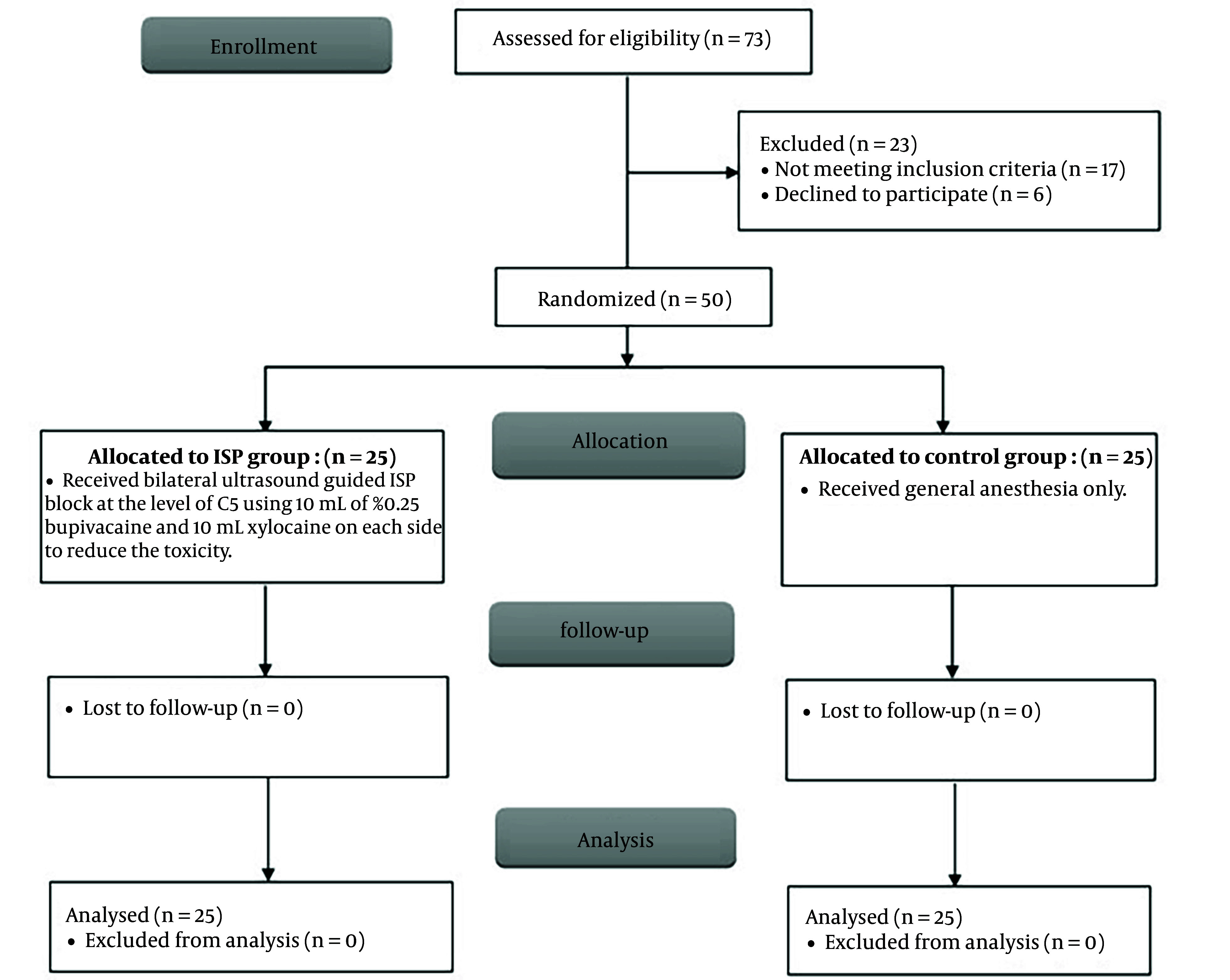
Consort flowchart

The study comprised 50 adult patients of either gender with ASA physical status I-II who were undergoing elective posterior cervical spine surgery. They were randomly divided into two equal groups. All patients were closely monitored, and a thorough statistical analysis was conducted (Figure 1). There were no statistically significant differences between the two groups in terms of demographic characteristics, including age, gender distribution, weight, height, and BMI (Table 1). 

**Table 1.** Demographic Data of the Studied Groups

Variables	ISP Group (n = 25)	Control Group (n = 25)	P-Value ^a^
**Age (y)**			0.478
Mean ± SD	51.32 ± 10.35	53.04 ± 6.10	
Range	27 - 60	35 - 60	
**Sex, No. (%)**			0.556
Male	17 (68)	15 (60)	
Female	8 (32)	10 (40)	
**Weight (kg)**			0.093
Mean ± SD	83.56 ± 11.17	88.8 ± 10.46	
Range	68 - 110	75 - 105	
**Height (cm)**			0.94
Mean ± SD	163.68 ± 6.69	163.56 ± 4.34	
Range	152 - 176	155 - 173	
**BMI (kg/m** ^**2**^ **)**			0.19
Mean ± SD	31.4 ± 5.37	33.36 ± 5.04	
Range	23.3 - 43.5	25.4 - 42.6	

^a^ Statistically significant as P-value < 0.05.

Table 2 reveals that intraoperative fentanyl consumption was significantly lower in the ISPB group compared to the control group (P = 0.022). As indicated in Table 3, both groups had similar preoperative VAS scores. However, the ISPB group exhibited significantly lower VAS scores than the control group at 1, 8, 12, and 48 hours postoperatively (P = 0.016, 0.009, 0.005, and 0.016, respectively).

**Table 2.** Intraoperative Fentanyl Consumption of the Studied Groups

Fentanyl Consumption (Mic)	ISP Group (n = 25)	Control Group (n = 25)	P-Value ^a^
**Median (IQR)**	100 (100 - 100)	100 (100 - 150)	0.022
**Range**	100 - 200	100 - 200	

^a^ Statistically significant as P-value < 0.05.

**Table 3.** Comparison of Pre and Postoperative Parameters Between ISP Group and Control Group ^a^

Variables	ISP Group (n = 25)	Control Group (n = 25)	P-Value
**Pre-operative VAS of the studied groups**	5 (4 - 7)	5 (4 - 5.5)	0.301
30 min	3 (2 - 5)	4 (3 - 5)	0.171
1 h	3 (2 - 4)	4 (3 - 6)	0.016 ^b^
2 h	3 (2 - 5.5)	4 (2.5 - 4)	0.953
4 h	4 (3 - 4.5)	4 (2.5 - 5)	0.558
6 h	4 (2 - 5)	4 (3 - 5)	0.458
8 h	3 (2 - 4)	4 (3 - 5)	0.009 ^b^
12 h	4 (2 - 4.5)	5 (3.5 - 6)	0.005 ^b^
18 h	4 (2.5 - 5)	4 (3 - 5)	0.466
24 h	4 (2 - 5)	5 (3.5 - 5)	0.164
36 h	5 (3.5 - 6)	5 (4 - 5.5)	0.921
48 h	4 (4 - 5)	5 (4 - 6)	0.016 ^b^
**Postoperative pethidine requirement**			
Need	5 (20)	16 (64)	0.002 ^b^
First dose (mg)	30 (30 - 45)	30 (30 - 40)	0.66
Total dose (mg)	100 (75 - 135)	100 (82.5 - 100)	0.905

Abbreviation: VAS, Visual Analogue Scale.

^a^Values are expressed as No. (%) or median (IQR).

^b^Statistically significant as P-value < 0.05.

**Figure 2.**
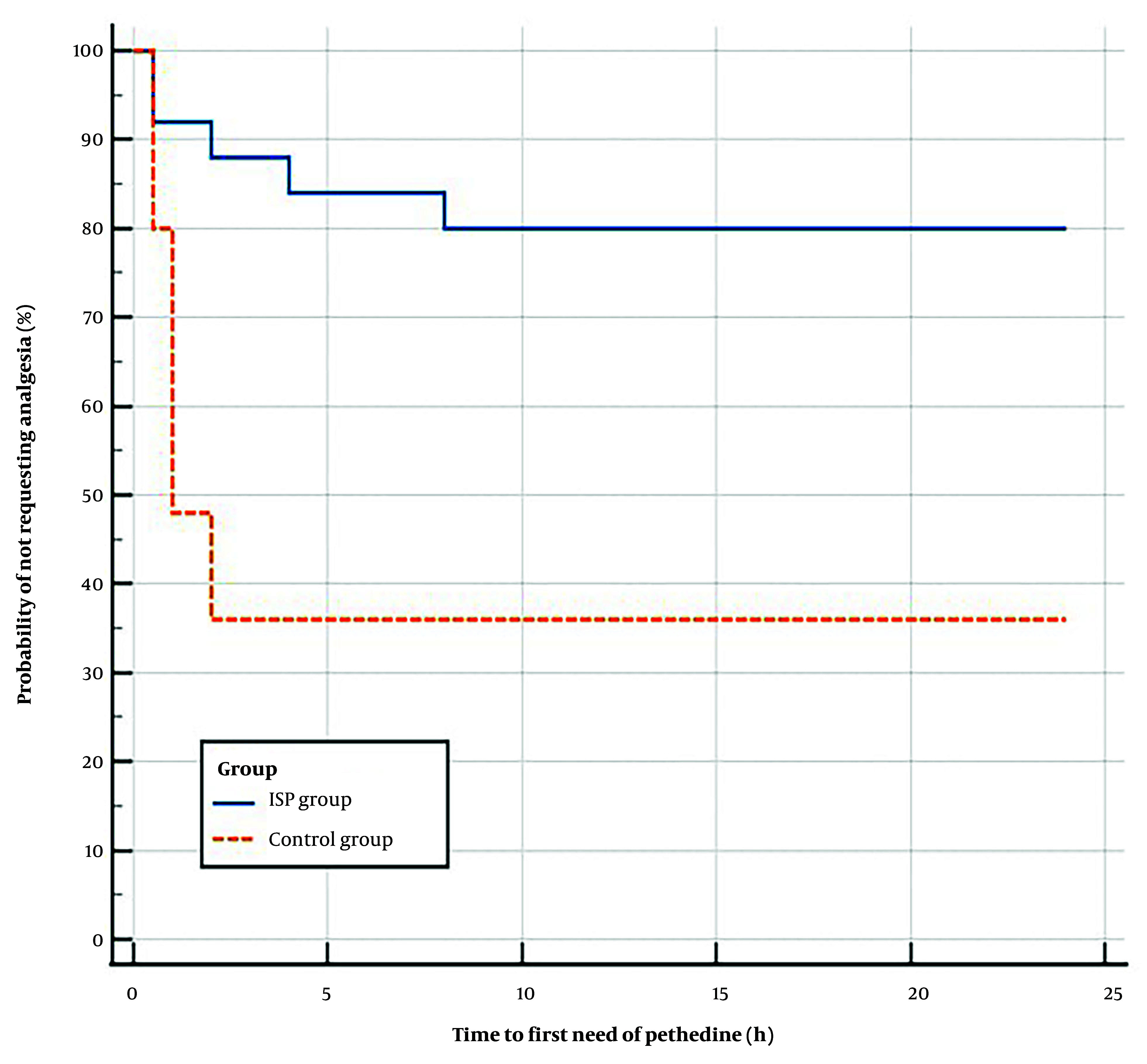
Kaplan-Meier curve analysis for time to first pethidine request

The ISPB group required significantly less pethidine for postoperative rescue analgesia compared to the control group (20% vs. 64%, P = 0.002), with no differences in the initial or total doses of pethidine between the 2 groups (Table 3). Kaplan-Meier curve and Log-rank test analysis showed that the ISPB group had a significantly longer median time to request pethidine postoperatively than the control group, with a hazard ratio of 0.215 (95% CI: 0.08 to 0.54, P = 0.001) (Figure 2). 

Regarding postoperative complications, Table 4 indicates no significant difference between the ISPB group and the control group. Patient satisfaction with the outcome was slightly higher in the ISPB group but not statistically significant compared to the control group. Surgeon satisfaction, however, was significantly higher with the results of the ISPB group than the control group (P = 0.003), as shown in Table 4. 

**Table 4.** Comparison of Complications and Patient Satisfaction Between ISP Group and Control Group ^a^

Variables	ISP Group (n = 25)	Control Group (n = 25)	P-Value
**Complications**			
Toxicity	0 (0)	0 (0)	-
Nausea	1 (4)	1 (4)	> 0.999
Vomiting	2 (8)	1 (4)	> 0.999
Pruritis	1 (4)	0 (0)	> 0.999
**Patient satisfaction**			0.125
Dissatisfied	7 (28)	8 (32)	
Fair	9 (36)	14 (56)	
Satisfied	9 (36)	3 (12)	
**Surgeon satisfaction**			0.003 ^b^
Not bad	0 (0)	5 (20)	
Fair	10 (40)	14 (56)	
Good	7 (28)	5 (20)	
Very good	8 (32)	1 (4)	

^a^ Values are expressed as No. (%).

^b^ Statistically significant as P-value < 0.05.

## 5. Discussion

Our study's analysis of postoperative rescue analgesia revealed that the demand for pethidine in the ISPB group was significantly lower than in the control group, although the total doses given to both groups were the same. The ISPB group showed a marked reduction in intraoperative fentanyl use compared to the control group. Both groups reported similar VAS scores prior to surgery. However, during the postoperative period, the ISPB group consistently reported significantly lower VAS scores at 1, 8, 12, and 48 hours after surgery. There was no statistically significant difference in postoperative complications between the ISP block group and the control group. Although patient satisfaction slightly increased in the ISPB group, this difference was not statistically significant. However, it is noteworthy that the ISPB group significantly surpassed the control group in terms of surgeon satisfaction with the surgical outcome. The ISPB is a novel ultrasound-guided technique that involves injecting a local anesthetic into the fascial plane between the semispinalis capitis and semispinalis cervicis muscles, targeting the dorsal rami of the cervical spinal nerves. This method effectively reduces postoperative pain (9). The sonographic target for the middle cervical plexus (MCP) block is the fascial plane between the multifidus cervicis and semispinalis cervicis muscles, while the target for the cervical interfascial plane (CIP) block is the plane between the multifidus cervicis and longissimus cervicis muscles. Their deep positioning within the posterior cervical region sometimes makes these structures difficult to differentiate using ultrasonography, particularly in older patients. The interscalene plexus (ISP) block, in contrast, aims for a shallower fascial plane than either the MCP or CIP block. Its more superficial location allows the ISP block to avoid issues such as dorsal artery perforation (10). Our research explored the analgesic effects of bilateral ISPB as an opioid-minimizing strategy for posterior cervical spine surgery. The results indicate that ISPB significantly reduces the total intraoperative fentanyl dose compared to the control group. Furthermore, patients receiving ISPB consumed significantly less postoperative morphine overall than those in the control group (1). Gerbershagen et al.'s study included patients undergoing spinal surgery under general anesthesia (2).

Patients who underwent surgeries involving 2 or more spinal levels consumed an average of 37.89 mg and 27.39 mg of morphine following their procedures, respectively. Furthermore, the occurrence of adverse symptoms such as nausea, vomiting, and sedation was notably lower in the ISPB group compared to the control group in this study. Additionally, patients in the ISPB group experienced significantly less pain during the first 12 hours post-surgery. Unlike the posterior cervical region described by Zhang et al. (11), a previous study (8) presented a modified and more superficial approach to the middle cervical plexus block (MCPB), where the local anesthetic is administered into a deeper layer between the multifidus and cervical spinal nerves but within a more reachable layer between the semispinalis cervicis and semispinalis capitis muscles (11). Ohgoshi and Kubo (12) documented the successful use of the Interscalene Brachial Plexus Block (ISPB) in a patient scheduled for cervical spine surgery. Compared to the MCP, the ISPB provides easier access and a clearer sonographic view, particularly in elderly patients with complex anatomical variations. Moreover, the ISPB may reduce the risk of accidental intrathecal injections that could complicate MCPB (12). Meng et al. (13) conducted a meta-analysis of 17 randomized controlled trials to compare the efficacy of epidural analgesia with intravenous analgesia following spine surgery. They concluded that epidural analgesia is an effective pain management strategy, enabling patients to use fewer opioids on the first postoperative day compared to control groups. However, the use of neuraxial techniques comes with a 15-fold increased risk of motor block, which could impede postoperative neurological monitoring and recovery. Additionally, there is a risk of dural puncture, which may result in the leakage of local anesthetic at the surgical site, leading to uneven tissue absorption. Furthermore, complications such as obstruction, displacement, and infection are frequently associated with the epidural catheter itself (14). The ISPB is limited to anesthetizing the dorsal rami of the spinal nerves, excluding the brachial plexus, due to the barrier created by the semispinalis capitis muscle, which prevents the spread of local anesthetic. However, evaluating the outcomes of postoperative neurosurgical procedures can be challenging due to motor paralysis caused by brachial plexus anesthesia during Erector Spinae Plane Blocks (ESPB) (15, 16). Cadaveric studies by Elsharkawy et al. (15) and Diwan et al. (17) have shown that local anesthetic can spread to the phrenic nerve following cervical ESPB, highlighting the importance for practitioners to be mindful of these potential outcomes (18). In contrast, the semispinalis capitis muscle in ISPB acts as a barrier, preventing local anesthetic from reaching the phrenic nerve (16). The posterior approach to cervical spine surgery facilitates efficient and straightforward management of the posterior neural elements and upper cervical vertebrae. However, the extensive midline incision, muscle retraction, mechanical injury caused by surgery, and the removal of bone and ligaments can lead to significant postoperative pain (19), with moderate to severe pain reported in up to 70% of cases.

Reports indicate that inadequate management of postoperative pain is associated with various surgical, autonomic, and metabolic complications, as well as higher incidences of patient complaints (2, 20). Although nonsteroidal anti-inflammatory drugs (NSAIDs) serve as an effective analgesic in the perioperative context and play a pivotal role in multimodal analgesia, their use in patients undergoing spinal surgery carries risks such as bone nonunion and postoperative bleeding (21). Nonetheless, the adverse impacts of NSAIDs can be mitigated by administering them in low doses for short durations or by choosing selective COX-2 inhibitors (22, 23). Furthermore, systematic reviews conducted by Zambouri (24) and Alboog et al. (25) have shown that the concerns regarding NSAID use in spinal surgery are based on evidence of low quality.

### 5.1. Strengths and Limitations

The strengths of this study are highlighted by its randomized controlled design, which reduces bias, and the explicit inclusion and exclusion criteria that aid in identifying a precise patient group. The study's adherence to ethical guidelines is evidenced by its ethical approval, registration with ClinicalTrials.gov, and a thorough informed consent process, showcasing a dedication to transparency and ethical practices. Furthermore, the calculation of the sample size, informed by a prior study and power analysis, underpins the statistical reliability of the findings. The use of well-defined outcome measures, including postoperative pethidine consumption, VAS scores, and surgeon satisfaction, offers meaningful insights into the study's results. Despite its strengths, the study has limitations that warrant consideration. The focus on a specific patient population and the single-center setting may restrict the generalizability of the findings. Additionally, the scarcity of research on the ISPB in cervical surgery presents a limitation, resulting in limited available information and highlighting the need for further investigation, particularly at the cervical level. This approach, however, enabled us to establish a comprehensive background for our research. Future studies should specifically focus on the ISPB to enhance our understanding of its clinical implications.

### 5.2. Conclusions

The ISP block proves to be an effective analgesic technique in posterior cervical spine surgery. It reduces opioid consumption, enhances pain control, and increases surgeon satisfaction without raising the risk of complications. This approach has the potential to improve postoperative care and patient outcomes within this surgical population.

## Data Availability

Data are available from the corresponding author upon reasonable request.

## Clinical Trial Registration Code:

NCT06003933.
